# Laparoscopic versus open liver resection for huge hepatocellular carcinoma (≥ than 10 cm): a multicenter propensity score-matched analysis from Eastern and Western referral centers

**DOI:** 10.1007/s00464-026-12597-9

**Published:** 2026-01-27

**Authors:** Gianluca Cassese, Fabio Giannone, Federica Cipriani, Antonio Cubisino, Bruno Branciforte, Alessandro Tropea, Fabio Benedetti, Fabrizio Romano, Salvatore Gruttadauria, Guido Torzilli, Mickael Lesurtel, Luca Aldrighetti, Ho-Seong Han, Patrick Pessaux, Fabrizio Panaro

**Affiliations:** 1 Division of Hepato-Pancreato-Biliary, Oncologic and Robotic Surgery, Department of Research and Innovation (DAIRI), Azienda Ospedaliero-Universitaria SS. Antonio e Biagio e Cesare Arrigo, Alessandria, Italy; 2 Division of Hepato-Pancreato-Biliary Surgery, Department of Surgery, Seoul National University Bundang Hospital, Seongnam, South Korea; 3 Department of Health Sciences, University of Eastern Piedmont “Amedeo Avogadro”, Azienda Ospedaliero-Universitaria SS. Antonio e Biagio e Cesare Arrigo, Alessandria, Italy; 4 Department of Visceral and Digestive Surgery, University Hospital of Strasbourg, Strasbourg, France; 5 Hepatobiliary Surgery Division, IRCCS San Raffaele Scientific Institute, Milan, Italy; 6 Department of HPB Surgery and Liver Transplantation, Beaujon Hospital, APHP, University of Paris Cité, Clichy, France; 7 Division of Hepatobiliary and General Surgery, Department of Surgery, Humanitas Clinical and Research Center - IRCCS, Humanitas University, Rozzano, Milan, Italy; 8 Department for the Treatment and Study of Abdominal Diseases and Abdominal Transplantation, IRCCS-ISMETT, UPMC (University of Pittsburgh Medical Center), Palermo, Italy; 9 Department of General Surgery, Unit of Hepatobiliary Surgery, IRCCS San Gerardo dei Tintori, Milano-Bicocca University, Monza, Italy; 10 Inserm, U1110, Institut de Recherche sur les Maladies Virales et Hépatiques, Université de Strasbourg, Strasbourg, France

**Keywords:** Huge hepatocellular carcinoma, Minimally invasive liver surgery, Hepatocellular carcinoma

## Abstract

**Background:**

There is still poor evidence about the safety and feasibility of minimally invasive liver surgery (MILS) for huge (> 10 cm) hepatocellular carcinomas (HCC). The aim of this study was to assess the short- and long- term outcomes of MILS versus open liver resection (OLR) for patients with huge HCC.

**Methods:**

Data regarding all consecutive patients undergoing liver resection for huge HCC were retrospectively collected from Asian (South Korean) and European (Italian and French) referral HPB centers. The cases were propensity score matched for age, center, extent of the resection, tumor size, and tumor number.

**Results:**

A total of 198 patients were included in the study. Before matching there were statistically significant differences in tumor size (*p* < 0.01) and rates of major hepatectomies performed (*p* = 0.03). After PSM two cohorts of 39 patients were obtained, with no statistically significant differences in all the compared preoperative characteristics. No significant differences were found in terms of major complications, in-hospital mortality, and operative time, between the matched cohorts. The median length of hospital stay was significantly lower after MILS (7 vs. 10 days, *p* < 0.01), as well as the median intraoperative estimated blood loss (500 ml vs 800 ml, respectively; *p* = 0.02) and the rates of intraoperative transfusions (25.6% vs 48.7%, respectively; *p* = 0.03). After a median follow-up of 52 months, there were no significant differences between OLR and MILS in median OS (44 vs. 93.6 months, respectively; *p* = 0.07). Median DFS was improved after MILS (49.8 vs. 7 months, respectively; *p* < 0.01).

**Conclusion:**

MILS for huge HCC can be safe and effective in selected cases in referral centers, being able to reduce intraoperative blood loss, and to shorten median hospital stay.

Hepatocellular carcinoma (HCC) is the most common primary liver cancer, being listed as the seventh most common cancer worldwide and the third leading cause of cancer-related death [1]. Despite both medical and surgical improvements, HCC prognosis is still poor, with 5 years overall survival (OS) as low as 20% [2]. Surgery represents the cornerstone treatment for early-stage HCC, leading to better survival outcomes, reaching up to 50–70% of 5 years OS. Ideally, liver transplantation (LT) is the best available therapy for HCC, aiming to treat both HCC and underlying chronic liver disease, but it must face the problems of organ shortage, with a consequent risk of dropout from waiting list and tumor progression [3]. Furthermore, the Milan criteria restrict LT in adults to patients with nodules smaller than 5 cm, or less than 3 lesions and each one not exceeding 3 cm, without vascular invasion, without extrahepatic involvement [4]. Therefore, liver resection still represents the most performed treatment for early stages.

HCC is often diagnosed in advanced stages, with large (> 5 cm) or huge lesions (> 10 cm) [5]. These patients cannot benefit from LT, neither can undergo thermal ablation due to the impossibility to achieve complete tumor necrosis of nodule larger than 3 cm [6]. Nonetheless, according to current guidelines, patients with a solitary HCC and preserved liver function can still benefit from liver resection, when preserving enough FRL to sustain liver metabolic activity [7]. Extended resections of a cirrhotic liver may be required for such cases, with the subsequent risk of postoperative morbidity and mortality, mainly related to post-hepatectomy liver failure (PHLF) [8]. In this setting, liver resection has shown survival advantages when compared to trans-arterial chemoembolization (TACE) [9]. In patients with huge HCC, the presence of extrahepatic collaterals is a limitation for achieving the complete embolization of the tumor [10]. An alternative to surgery in such cases may be trans-arterial radioembolization (TARE). However, there is no evidence about any advantage of TARE on surgery in huge HCC, while the risk of pulmonary complications of the radioembolization in this setting is non-neglectable.

Last European guidelines about HCC treatment recommend the use of minimally invasive liver surgery (MILS) for early staged tumors, in light of a reduced risk of decompensation in case of cirrhosis, enhanced recovery, and less intraoperative blood loss, while achieving equivalent long-term oncological outcomes than open liver resection (OLR) [7, 11, 12]. Nonetheless, several difficult situations still limit the universal adoption of MILS for HCC treatment [13]. Despite few small-sized retrospective studies showed some encouraging data about the outcomes of MILS for huge HCC, this setting certainly represents a challenging and debated indication for MILS [14].

The aim of this study is to analyze postoperative outcomes of MILS for patients with huge HCC.

## Methods

### Patients and data

Data from consecutive patients undergoing laparoscopic liver resection for large HCC from January 2010 to September 2022 at 7 tertiary referral HPB centers across Europe and Asia were retrospectively collected from prospectively established databases. The inclusion criteria were as follows: male or female patients aged ≥ 18 years old; one or more nodules with histopathological confirmation of HCC ≥ 100 mm; no extrahepatic disease; adequate FRL. The exclusion criteria were as follows: liver resection extended to other abdominal organs other than gallbladder; histological finding of combined HCC and cholangiocarcinoma. According to the different surgical procedures, patients were divided into two groups as follows: the MILS group and the OLR group. Primary outcomes were postoperative results, while survival outcomes constituted secondary endpoints.

This study was conducted according to the Strengthening and Reporting of Observational Studies in Epidemiology (STROBE) guidelines of the EQUATOR network. Informed consent was obtained prior to every surgical procedure [15]. All subjects gave their informed consent, and the study was conducted in accordance with the Declaration of Helsinki after institutional review board approval.

### Preoperative management

The choice of the therapeutic management of each of the included patients was taken after a multidisciplinary team meeting including hepatologists, oncologists, and radiologists. Preoperative and postoperative management, as well as surgical procedures, were performed by the same team in the same facility. Preoperative evaluations were similar in both groups and included routine blood tests, liver function, coagulation examinations, alpha-fetoprotein (AFP) levels, and triphasic enhanced computed tomography (CT) and/or magnetic resonance imaging (MRI). The definition of resectable HCC was based on multidisciplinary team decision, according to comprehensive evaluation of liver function test, remnant liver volume, and liver compensation status [16].

All the included centers are referral HPB centers performing both MILS and OLR. After surgeons fully informing the patients about the pros and cons of the two approaches, the final decision was made by surgeons’ and patients’ preferences. The proximity of major vessels, with the subsequent risk of ischemia of the remnant liver or R1 resection, as well as the size of the lesions, were the main factors to decide the type and the extent of the resection. A major hepatectomy was carried out in cases of proximity of the first- and second-order vascular branches and/or presence of huge lesions occupying a hemiliver.

### Postoperative management and follow-up

Postoperative follow-up data were analyzed. Postoperative complications were classified according to the Clavien–Dindo classification, with severe complications defined as Clavien–Dindo ≥ 3. PHLF, post-hepatectomy bile leakage (PHBL), and post-hepatectomy hemorrhage (PHH) were diagnosed and classified according to the International Study Group of Liver Surgery (ISGLS) guidelines. Ascites was defined according to the International Ascites Club definition.

All patients were examined in outpatient clinics within 1 month after discharge, undergoing clinical, biological, and imaging evaluations every 3–4 months after discharge for the first 2 years, according to the oncological protocols. Following controls were scheduled every 6 months if no relapse was found. In case of tumor recurrence, the case was re-examined by a multidisciplinary team (MDT) with the aim of carrying out curative treatment as much as possible. The first therapeutic strategy for localized recurrent HCC was repeat hepatectomy, according to previous studies that have shown the same OS and DFS as primary liver resection. In cases where liver resection was not indicated because of liver, as well as tumor or patient status, other locoregional therapies represented the second choice.

### Statistical analysis

Continuous data are expressed as mean and standard deviation (SD) or median and inter-quartile range (IQR), depending on whether they had a normal distribution or not. Group comparisons were performed using Student’s *t* test or Wilcoxon's rank test, depending on the distribution of the variable. Categorical data are expressed as frequencies and associated percentages. Comparisons between groups were performed using Pearson's chi-squared test or Fisher’s exact test, depending on the expected value of the variable of interest. Overall and recurrence-free survival analyses were performed using the Kaplan–Meier method to calculate the median and 95% confidence interval (CI), and comparisons were performed using the log-rank method. The median follow-up was analyzed using the inverse Kaplan–Meier method. To compare the two cohorts by minimizing the selection bias, a propensity score-matched (PSM) analysis was performed with a caliper width of 0.50, obtaining a one-to-one match, and excluding patients in whom the PSM was not applicable. The model was based on logistic regression, using the single nearest neighbor matching method without replacement. The two groups were matched for: age, center, cirrhosis, tumor size, extent of the resection. P values < 0.05 were considered statistically significant. All statistical analyses were performed using SPSS software version 26 (IBM SPSS Inc. Chicago, IL).

## Results

### Patients and characteristics

One hundred and ninety-seven consecutive patients undergoing liver resection for huge HCC were included, of which 158 (80.2%) were laparotomic and 39 (19.8%) we minimally invasive.

Before matching, there were statistically significant differences between the 2 groups in tumor sizes (median 130 mm in OLR [60] vs. 105 mm in MILS [17]) and in the rates of major hepatectomies (74.1% in OLR [*n* = 117] vs 56.4% in MILS [*n* = 22]).

After propensity score matching, two cohorts of 39 patients were obtained. There were no statistically significant differences between the cohorts. Median age was 64 years (IQR = 25) in OLR patients vs. 63 years (IQR = 20) in MILS patients (*P* value = 0.80). Underlying liver cirrhosis was present in 17 patients undergoing OLR (43.6%) vs 15 in MILS group (38.5%) (*p* = 0.64). Median tumor size was 110 mm (IQR = 17) vs. 105 mm (IQR = 21), respectively) (*P* value = 0.18), with 29 patients undergoing open major hepatectomy and 22 patients undergoing minimally invasive major liver resection (*p* = 0.10).

All preoperative patients’ and tumors’ characteristics are shown in Table 1.

**Table 1:** Preoperative patients’ and tumors’ characteristics before and after propensity score matching

	Before matching			After matching		
	Open (*n* = 158)	Minimally invasive (*n* = 39)	*P* value	Open (*n* = 39)	Minimally invasive (*n* = 39)	*P* value
Age, median (IQR)	64 (18.6)	63 (20.7)	0.98	64 (25)	63 (20)	0.80
Female sex, *n* (%)	45 (28.5)	6 (15.4)	0.09	3 (7.7)	6 (15.4)	0.28
BMI, median (IQR)	24.6 (4.6)	25.6 (2.7)	0.22	24.4 (4)	25.6 (2.7)	0.15
ASA score, * n* (%)						
1	26 (16.4)	2 (5.1)	0.15	0 (0)	2 (5.1)	0.21
2	74 (46.9)	24 (61.6)		21 (53.8)	24 (61.6)	
3	54 (34.2)	13 (33.3)		16 (41.1)	13 (33.3)	
4	4 (2.5)	0 (0)		2 (5.1)	0 (0)	
Cirrhosis, * n* (%)	39 (24.7)	15 (38.5)	0.08	17 (43.6)	15 (38.5)	0.64
Child, * n* (%)						
A	36 (92.3)	14 (93.4)	0.89	12 (70.6)	13 (86.6)	0.23
B	3 (7.7)	1 (6.6)		5 (19.4)	2 (13.4)	
Tumor size, median (IQR)	130 (60)	105 (21)	** < 0.01**	110 (17)	105 (21)	0.18
Tumor number, * n* (%)						
1	138 (87.3)	35 (89.8)	0.64	35 (89.8)	35 (89.8)	1.00
2	15 (9.5)	2 (5.1)		2 (5.1)	2 (5.1)	
≥ 3	5 (3.2)	2 (5.1)		2 (5.1)	2 (5.1)	
Major hepatectomy, * n* (%)	117 (74.1)	22 (56.4)	**0.03**	29 (74.3)	22 (56.4)	0.10
AFP, median (IQR)	39 (839)	20 (410)	0.46	10 (287)	20 (410)	0.77
Microvascular invasion, * n* (%)	103 (65.1)	22 (56.4)	0.23	26 (66.6)	22 (56.4)	0.35
Satellite nodules, * n* (%)	65 (41.1)	9 (23.1)	**0.03**	8 (20.5)	8 (20.5)	0.90
Grading, * n* (%)						
1	38 (24)	10 (25.6)	0.75	5 (12.8)	10 (25.6)	0.09
2	71 (44.9)	14 (35.8)		10 (25.6)	14 (35.8)	
3	49 (31.1)	15 (38.4)		24 (61.5)	15 (38.4)	

Bold indicates statistically significant values

*IQR* inter-quartile range, *BMI* body mass index, *ASA* American Society of Anesthesiologist classification, *AFP* alpha-fetoprotein

### Perioperative outcomes

The matched cohorts showed no significant differences in operative time, as well as in postoperative PHH, PHLF, severe complication rates, and in-hospital mortality. MILS cohort showed reduced median estimated blood loss (EBL) (500 ml vs. 800 ml, respectively; *P* value = 0.02), lower rates of intraoperative transfusions (25.6% vs. 48.7%, respectively; *P* value = 0.03), and shorter median length of stay (7 days vs. 10 days, respectively; *P* value < 0.01). OLR had shorter Pringle maneuver duration (0’ vs.17’, respectively; *P* value = 0.04). Perioperative outcomes comparison after propensity score matching is available in Table 2.

**Table 2:** Perioperative outcomes of matched cohorts

	Open (*n* = 39)	Minimally invasive (*n* = 39)	*P* value
Operative time, median (IQR)	300 (145)	269 (160)	0.45
Pringles maneuver duration, median (IQR)	0 (20)	17 (45)	**0.04**
EBL, median (IQR)	800 (800)	500 (700)	**0.02**
Blood transfusions, * n* (%)	19 (48.7)	10 (25.6)	**0.03**
PHLF, * n* (%)	3 (7.7)	2 (5.1)	0.64
PHH, * n* (%)	1 (2.6)	0 (0)	0.31
Severe complications, * n* (%)	8 (20.5)	5 (12.8)	0.36
Length of stay, median (IQR)	10 (6)	7 (7)	** < 0.01**
In hospital mortality, * n* (%)	1 (2.6)	1 (2.6)	1.00

Bold indicates statistically significant values

*IQR* inter-quartile range, *EBL* estimated blood loss, *PHLF* post-hepatectomy liver failure, *PHH* post-hepatectomy hemorrhage

### Survival outcomes

**Fig. 1:**
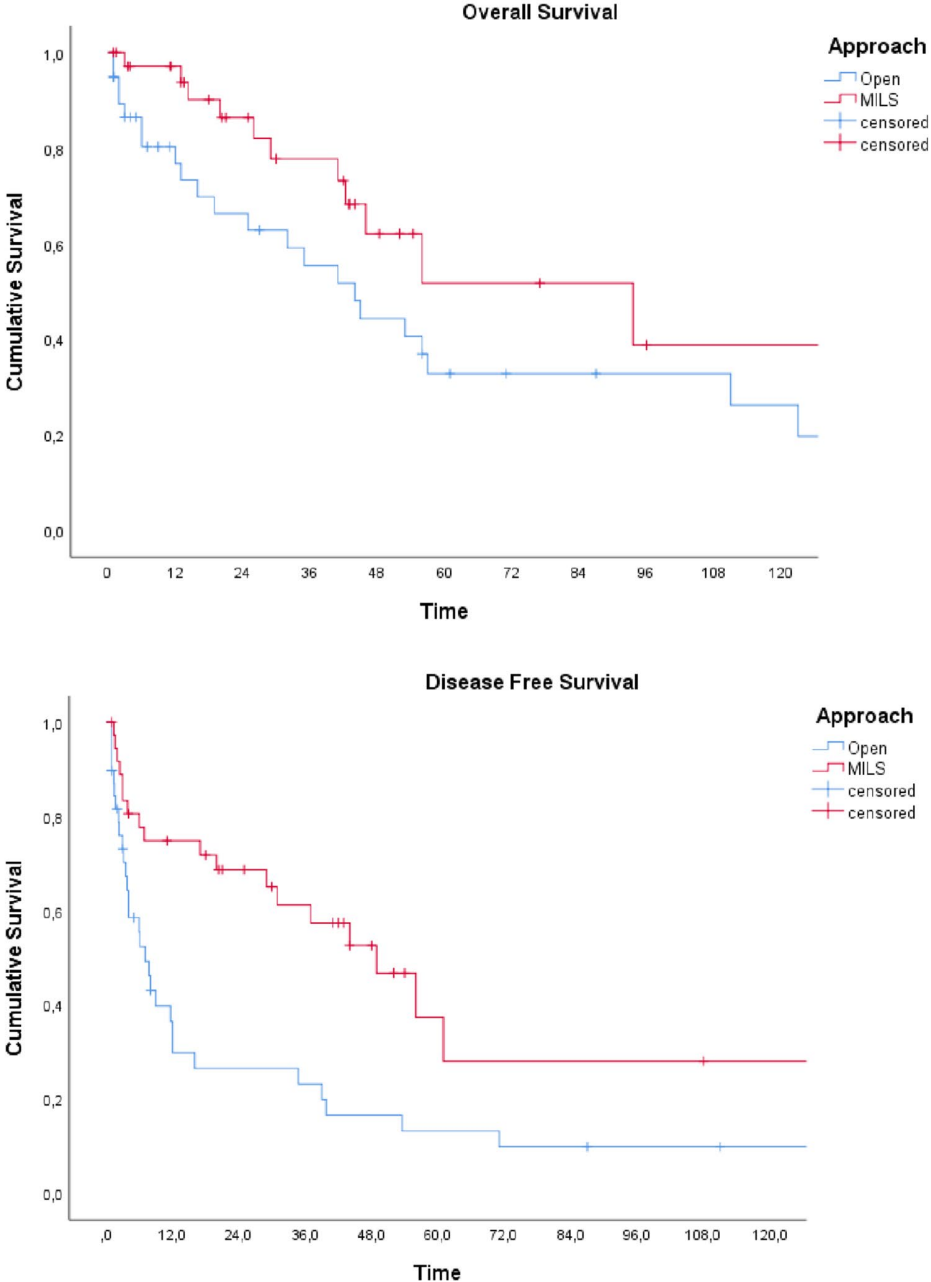
Survival outcomes after open liver resection (blue) and minimally invasive liver surgery (red)

After a median follow-up of 52 months (95% CI 40–66), there were no statistically significant differences in median OS, despite a tendency to an improved survival after MILS (44 months after OLR [95% CI 27.6–60.4] vs. 93.6 months after MILS [95% CI 34.4–153.8]; *P* value = 0.07). Median DFS was significantly better after MILS (49.8 months after MILS [95% CI 30.3–67.5] vs.7 months after OLR [95% CI 4.3–9.7]; *P* value < 0.01). Survival outcomes are reported in Fig. 1.

## Discussion

This study represents the first multicenter study investigating the short- and long-term outcomes of MILS for huge HCC, with the largest sample size in literature.

Previous studies investigating the role of laparoscopic liver resection for large liver cancer showed how it can be performed safely, concluding that tumor size does not affect both short- and long-term outcomes [18, 19]. Nonetheless, MILS for large tumors may be challenging because of limited surgical view, limited possibility of handling the underlying fibrotic or cirrhotic liver, and the proximity of such lesions to vascular and biliary structures. Indeed, tumor size is one of the main parameters of the most used difficulty scores for MILS, and an interesting recent study reports a correlation between technical difficulty and long-term results after minimally invasive liver resection [16, 20]. Therefore, MILS for huge HCC has to be considered even more technically demanding and is currently performed only by experienced surgeons in referral HPB centers.

In our study, after propensity score matching the data from consecutive patients with huge HCC from referral HPB center, MILS showed improved postoperative outcomes for patients with huge HCC, thanks to reduced median EBL, lower rates of intraoperative transfusions, and shorter median length of stay. This result is coherent with previous literature that has reported reduced bleeding for large HCC undergoing laparoscopic liver resection [21]. Furthermore, intraoperative bleedings have previously been related to impaired long-term oncological outcomes in HCC patients [17]. Therefore, efforts to minimize both EBL and intraoperative transfusions during hepatic resection are important for improving long-term prognosis in HCC patients. On the other hand, OLR was associated with shorter Pringle maneuver duration. However, despite longer, the median time was only 17’ in MILS patients that is a relatively short duration in line with previous literature [22]. To note the indications and the threshold for Pringle maneuver application was not a priori established: every surgeon decided based on the specific case and his experience or preference.

Similarly, the reduced length of hospital stay after MILS has been often reported in various situations, including large HCC or elderly patients [23, 24]. Indeed, a previous single-center study from Seoul National University Bundang Hospital already showed a reduced stay after laparoscopic liver resection for huge HCC [25]. These results could be related to reduced postoperative pain, early ambulation, and early recovery of gastrointestinal function [26]. Moreover, a reduced length of hospital stay could led to a significant reduction in the economic costs related to HCC treatment. However, the economic aspects were not within the aims of our study: further efforts should investigate such an important field.

When focusing on long-term outcomes, liver resection has been shown to be the best treatment for huge HCC, when compared to other strategies [27]. Long-term recurrence is the main problem to face in these patients, and several prognostic risk factors have been identified, such as T4 status, macrovascular portal invasion, and the use of intraoperative transfusion by Yamashita and al., or serum alpha-fetoprotein ≥ 100 ng/mL, hypermetabolic uptake on positron emission tomography, satellite nodules, and microvascular invasion [28]. In case of recurrence, timely and aggressive treatment is able to significantly improve long-term survival of HCC patients, with recurrent surgery always to be chosen in the optic of a hierarchic strategy and a personalized management of HCC patients [29]. Within the entire cohort of the patients included in our study the median OS was 56 months [95% CI 41.6–70.3]. Such encouraging results are in line with previous literature and confirm the leading role of surgery for huge HCC patients. Interestingly, both median OS and DFS were improved in the MILS group. However, such results must be interpreted with caution, since many prognostic factors, such as some pathological characteristics of the tumors were not analyzed, because of some missing data. Further appropriate studies should investigate this aspect.

This study has some other limitations. Firstly, its retrospective nature may lead to selection bias. Nonetheless, a propensity score matching was applied to reduce them as much as possible. Furthermore, all consecutive patients meeting the selection criteria were included. Secondly, the multicenter nature may add some heterogeneity to analysis and results, since treatments and managements may somehow vary among centers. As previously stated, the survival outcomes were not analyzed through a multivariable analysis due to the lack of some important additional data.

Finally, this study may play a role in confirming the role of MILS for the surgical treatment of huge HCC. Indeed, the lack of large sample sized studies, as well as propensity score-matched analysis, has lead many centers to consider huge HCC not indicated for MILS. If further studies will confirm our results, huge HCC may be considered safe and feasible for a minimally invasive approach in referral centers, and it may be included in current recommendations.

## Conclusion

MILS is a safe option for huge HCC, in selected cases and in referral centers. In this setting, it may reduce intraoperative bleeding and postoperative length of hospital stay, while ensuring good long-term oncological outcomes.
